# Mental health symptoms among those affected by Huntington's disease: A cross‐sectional study

**DOI:** 10.1002/brb3.2954

**Published:** 2023-03-06

**Authors:** Sarah Gunn, Maria Dale, Noora Ovaska‐Stafford, John Maltby

**Affiliations:** ^1^ Centre for Psychological Health and Development, Psychology and Vision Sciences, College of Life Sciences University of Leicester Leicester UK; ^2^ Mill Lodge Huntington's Disease Inpatient and Community Service Leicestershire Partnership NHS Trust Leicester UK; ^3^ Greenwich Neurological Rehabilitation Team Oxleas NHS Foundation Trust London UK

**Keywords:** anxiety, apathy, Enroll‐HD, Huntington's disease, orientation

## Abstract

**Background:**

Although cognitive and motor symptoms of Huntington's disease (HD) are associated with disease progression, the underlying causes of psychological symptoms are not as clearly understood. Recent evidence suggests that some mental health difficulties experienced by people with HD are shared by noncarriers within HD families. Accordingly, there is a need to evaluate potential systemic contributors to HD mental distress, to support meaningful interventions for psychological symptoms in people with HD and affected families.

**Method:**

We used short‐form Problem Behaviors Assessment mental health symptom data from the international Enroll‐HD data set to characterize mental health symptoms across eight HD groups: Stages 1–5, premanifest and genotype‐negative individuals, and family controls (*n* = 8567) using chi‐square analysis with post hoc comparisons.

**Results:**

We identified that people with later‐stage HD (Stages 2–5) had significantly higher apathy, obsessive–compulsiveness, and (from Stage 3) disorientation than the remaining groups at a medium effect size, and that these findings largely held across three measure administrations over time.

**Conclusions:**

These findings highlight the critical symptoms in manifest HD from Stage 2 onward, but also demonstrate that crucial symptoms such as depression, anxiety, and irritability are present across HD‐affected groups (including noncarriers of the gene expansion). The outcomes highlight a need for specific clinical management of later‐stage HD psychological symptoms, and for systemic support across affected families.

## Graphical abstract

Recent evidence suggests that some mental health difficulties experienced by people with HD are shared by noncarriers within HD families. Accordingly, we characterized mental health symptoms across eight HD groups: Stages 1–5, premanifest and genotype‐negative individuals, and family controls (*n* = 8567). People with later‐stage HD had significantly higher apathy, obsessive–compulsiveness, and disorientation, but crucial symptoms such as depression, anxiety, and irritability were present across HD‐affected families.

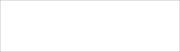

## 1 INTRODUCTION

Huntington's disease (HD) is an incurable autosomal dominant neurodegenerative disorder, arising from an expansion of cytosine–adenine–guanine (CAG) trinucleotide repeats on the short arm of chromosome 4. It typically arises in midadulthood (35–45 years), resulting in significant functional decline, and is fatal within 15–20 years of motor onset (Walker, 2007). It causes motor impairment, cognitive deterioration, and changes in emotions and behavior, with the latter often emerging a decade or more prior to diagnosable motor symptoms and presenting increasingly significant challenges to mental health as the condition progresses (Duff et al., 2007; Paulsen, 2011).

Although neural degeneration is believed responsible for many physical and psychological symptoms of HD (Martinez‐Horta et al., 2016), there has been less consideration of psychosocial contributors to HD‐related mental health. In particular, given the genetic nature of HD, symptoms occur within a family context, and there are systemic stressors affecting those with and without the gene expansion. Developing our understanding of these less‐examined factors underlying HD distress is crucial for informing interventional approaches. In this study, we seek to characterize key mental health symptoms arising across the time course of HD (premanifest and Stages 1–5). We also compare these symptoms with people affected by HD who do not carry the gene expansion (genotype‐negative and family control individuals), to determine the contribution of systemic factors to HD‐related distress. Exploring these issues will contribute to evidence‐based therapies for specific needs in particular HD groups, including interventions to systemically support HD families.

### 1.1 Cognitive and emotional changes in HD

Clinical diagnosis of “manifest” HD is based on the presence of motor signs such as chorea, rigidity, and bradykinesia. However, other changes begin up to 15 years before motor symptom onset. These include cognitive changes in memory, executive function, information processing, and emotional lability, and mental health symptoms such as depression, anxiety, irritability/anger, obsessive–compulsive behaviors, apathy, perseveration, and (less commonly) psychosis (Rickards et al., 2011; van Duijn, 2017; van Duijn et al., 2014, 2007). Although motor changes are viewed as the most characteristic symptoms of HD, both people with HD (pwHD) and their families report that mental health is a primary issue in care (Mahmood et al., 2022; Smith et al., 2015), and mental distress has been demonstrated throughout HD families, including genetically unaffected individuals (Achenbach & Saft, 2021; Maltby et al., 2021).

The neurodegeneration of striatal and cortical circuits is believed to be responsible for emotional and behavioral symptoms in pwHD (Martinez‐Horta et al., 2016). Suggested mechanisms include cingulate cortex degeneration affecting depressive symptoms, emotional recognition and visual working memory, and primary motor cortex cell loss affecting motor function (Hobbs et al., 2011; Thu et al., 2010). Microstructural changes in frontal, cingulate cortical, insula, and cerebllar regions have also been linked with subthreshold depressive symptoms (Sprengelmeyer et al., 2014). However, there is considerable variation in the presentation of the emotional and behavioral symptoms among pwHD and most of these symptoms (anxiety, depression, irritability/anger, and psychosis) have been reported as either unlinked or weakly linked to progression of cognitive and motor changes (Paulsen et al., 2001; Van Duijn et al., 2013; Zappacosta et al., 1996), except for apathy that carries a demonstrated link with HD progression (Martinez‐Horta et al., 2016; van Duijn et al., 2014).

### 1.2 Psychosocial contributors to HD symptoms

An alternative approach suggests a psychosocial contribution to HD‐related mental health, alongside biological neurodegeneration. The impact of HD on families can significantly affect mental health across gene‐expansion carriers and noncarriers. For example, both groups typically experience disrupted family dynamics, loss of relatives to HD, and changes to expectations about family roles and the future (Berrios et al., 2002; Brouwer‐DudokdeWit et al., 2002; Yu et al., 2019). There may also be fears about passing on HD to children, worries about care needs, disability and death in the future, and hypervigilance to potential signs of HD progression (Mahmood et al., 2022; Tibben et al., 1997; Wieringa et al., 2021). “Genotype‐negative” individuals who were genetically at risk but have tested negative may also continue to experience distress and intrusive thoughts relating to HD (Tibben et al., 1997). Such challenges may generate depression, anxiety, frustration, and apathy across HD‐affected families. Exploring contributions from these systemic/psychosocial factors could affect how such difficulties are understood, evaluated, and treated, and whether current theory and interventions adequately consider all contributors to HD‐related mental health.

Any potential impacts of social influences on HD‐related mental health symptoms can be explored by comparing pwHD (manifest and premanifest) against noncarriers who may be psychologically and socially affected by HD‐related stressors (family controls who were not at genetic risk, and genotype‐negative individuals who were at risk but tested negative). This enables the identification of symptoms unique to pwHD and those shared by other relevant HD populations. One recent exploration (Maltby et al., 2021) of underlying structures of psychological distress in HD populations identified four latent factors across manifest and premanifest HD, family control, and genotype‐negative groups, comprising symptoms relating to depression, anxiety, temper, and self‐harm. No significant differences in anxiety levels were identified between pwHD and control groups, and few mental health differences were found between people with manifest HD and family controls (although the manifest group reported higher depression, temper, and self‐harm than the genotype‐negative group). Overall, the findings suggested some important commonalities and differences in the structure and severity of mental health symptoms between pwHD and associated populations. However, these findings explored commonalities across broad domains (depression, anxiety, irritability), rather than considering specific clinical symptoms. Individual symptoms such as obsessive–compulsive behaviors, apathy, disorientation, perseveration, and psychosis may reflect key cognitive changes in memory, executive function, information processing, and emotional lability resulting from HD progression, and consequently exploration between HD‐affected groups at a symptom level is warranted.

### 1.3 Study aims

To improve our understanding of potential mechanisms underlying HD distress and to inform approaches to medical, pharmaceutical, and psychological interventions, it is important to characterize patterns of mental health symptoms across pwHD and related populations. To this end, we used the international Enroll‐HD data set to evaluate type and severity of mental health symptoms across HD stages and in related groups. We aimed to identify mental health symptoms unique to HD, and when in the disease course these occur, as well as examining which symptoms are shared within wider HD family systems. Importantly, we also evaluated replicability of findings across three timepoints, which is rare in the literature.

## 2 METHOD

### 2.1 Sample

Anonymized data were obtained from the international Enroll‐HD observational HD study (Enroll‐HD, RRID:SCR_023300), and approved by their Scientific and Bioethics Advisory Committee. The data that support the findings of this study are available from Enroll‐HD. Restrictions apply to the availability of these data, which were used under license for this study. Additional consent from participants for this study was not required. Males and females are eligible for inclusion. Individuals are excluded if they demonstrate choreic movement disorders but have tested negative for HD.

From an initial sample of 8714 participants who had complete date for three annual PBA‐s administrations, respondents were included at Time 1 (*n* = 8567) who had a completed short‐form Problem Behaviors Assessment (PBA‐s). Participants were from Europe (*n* = 4630), Northern America (*n* = 3503), Australasia (*n* = 323), and Latin America (*n* = 51). Participants were grouped into manifest HD (who have passed the point of motor onset of symptoms), premanifest HD (people who carry the expanded gene but have yet to develop motor symptoms of HD), genotype‐negative (people who were at risk, but tested negative), and family control subsamples (who were never at genetic risk but are part of an HD‐affected family) (see Table 1 for frequency data).

**TABLE 1:** Sample sizes by total and subsamples with gender and mean age, motor score, CAG, and mean age of formal diagnosis (for manifest HD group only) statistics for the three administration times

Administration		Manifest HD	Premanifest HD	Genotype‐negative	Family controls	Total sample
Time 1	Total *n* (male, female)	4630 (2277, 2353)	1845 (723, 1122)	1084 (362, 722)	1008 (446, 561)	8567 (3808, 4758)
	Mean age in years (SD)	52.88 (12.61) 9 did not disclose age	40.43 (11.94) 1 did not disclose age	42.96 (14.32) 3 did not disclose age	54.14 (12.43) 1 did not disclose sex 1 did not disclose age	49.09 (13.87) 1 did not disclose sex 14 did not disclose age
	Mean motor score (SD)	38.69 (21.19) 25 did not report	3.53 (5.28) 6 did not report	1.97 (3.97) 6 did not report	1.61 (2.90) 3 did not report	22.09 (23.98) 40 did not report
	Mean CAG (SD)	43.98 (3.76) 12 did not report	42.43 (2.78)	20.26 (3.66)	20.07 (3.42)	37.82 (10.66) 12 did not report
	Mean age (SD) of formal diagnosis of HD	48.31 (12.61) 132 did not report				
Time 2	Total *n* (male, female)	2656 (1315, 1341)	925 (356, 569)	546 (177, 369)	517 (226, 290)	4644 (2074, 2569)
	Mean age in years (SD)	53.69 (12.20) 3 did not disclose age	41.91 (12.12)	46.00 (14.52)	56.45 (12.01) 1 did not disclose sex 1 did not disclose age	50.74 (13.49) 1 did not disclose sex 4 did not disclose age
	Mean motor score	39.92 (21.43) 14 did not report	3.58 (4.78) 5 did not report	1.69 (2.89) 1 did not report	1.81 (3.79) 1 did not report	23.93 (24.72) 21 did not report
	Mean CAG (SD)	43.93 (3.68) 6 did not report	42.17 (2.67)	20.15 (3.62)	19.85 (3.34)	38.09 (10.48) 6 did not report
	Mean age (SD) of formal diagnosis of HD	48.32 (12.06) 70 did not report				
Time 3	Total *n* (male, female)	967 (496, 471)	358 (134, 224)	245 (68, 177)	275 (119, 155)	1845 (817, 1027)
	Mean age in years (SD)	54.25 (11.81) 1 did not disclose age	42.61 (12.03)	48.70 (14.01)	57.85 (11.82) 1 did not disclose sex	51.79 (13.20) 1 did not disclose sex 1 did not disclose age
	Mean motor score	39.97 (20.85) 13 did not report	3.84 (4.69) 1 did not report	1.79 (3.52) 1 did not report	1.71 (2.95) 2 did not report	22.10 (24.15) 17 did not report
	Mean CAG (SD)	43.92 (3.61) 3 did not report	42.20 (2.68)	19.94 (3.28)	19.65 (3.44)	36.77 (11.20) 3 did not report
	Mean age (SD) of formal diagnosis of HD	47.86 (11.77) 18 did not report				

To evaluate replicability, we also extracted data for the next two annual PBA‐s administrations for this sample (Time 2: *n* = 4644; Time 3: *n* = 1845). Participants for Time 2 (*n* = 4644) and Time 3 (*n* = 1845) were lower due to some individuals not participating in second and third annual assessments.

### 2.2 Measures

The PBA‐s is the recommended screening tool for behavioral symptoms in HD (McNally et al., 2015). It comprises an 11‐item semistructured interview describing HD‐related mental health symptoms and behavioral difficulties (depression, anxiety, irritability, aggression, apathy, obsessive–compulsive behaviors, perseveration, paranoid thinking/delusions, hallucinations, and disorientation). The scale is administered by a trained interviewer, who rates items on 5‐point scales for severity (0 = *absent* to 4 = *severe*) and frequency (0 = *never or almost never* to 4 = *daily/almost daily for most or all of the day*). Overall item scores are calculated by multiplying frequency and severity, with scores ranging from 0 to 16. Item scores of 2 or higher indicate at least “mild” presence of that symptom. For our analyses, we used the presence or absence of symptoms for the 11 symptom domains (0 = not present, 1 = present).

We assigned participants to the manifest HD, premanifest HD, genotype‐negative, or family control subsamples, and subdivided the manifest group into five subgroups based on Total Functional Capacity (TFC) scale scores (Shoulson & Fahn, 1979). This is a frequently used assessment of function in HD (Paulsen et al., 2010). Like the PBA‐s, the TFC scale is completed via a brief interview in which the pwHD is assessed for functional abilities around occupation, finances, domestic chores, activities of daily living, and care needs, with scores on each item ranging from 0 (“unable”) to 3 (“normal”). Item scores are summed, with possible totals ranging from 0 to 13. Higher scores indicate higher capacity for functioning. Scores from 11 to 13 represent Stage 1 (least severe); 7–10 represent Stage 2; 3−6 represent Stage 3; 1−2 represent Stage 4; and 0 represents Stage 5 (most severe). Therefore, in total, we used eight subsamples: manifest HD Stages 1 through to 5, premanifest HD, genotype‐negative, and family control. HD stage is reconfirmed independently at each study visit. For subsample sizes at the three PBA‐s administration times, see Tables 2, 3, 4.

**TABLE 2:** Frequency (%), chi‐square, effect size, and post hoc comparison statistics for mental health symptoms by subsample at Time 1 (*n* = 8567)

		Family controls	Genotype‐negative	Premanifest	Stage 1	Stage 2	Stage 3	Stage 4	Stage 5			
		*n* = 1008	*n* = 1084	*n* = 1845	*n* = 1367	*n* = 1700	*n* = 1110	*n* = 371	*n* = 82			
		Symptoms % (adjusted residual *z*‐score)								*χ* ^2^	*w*	Power
1. Depression	+	30.1% (−5.4)^**^	23.4% (−10.4)^**^	37.6% (−0.1)	41.6% (3.2)	45.8% (7.7)^**^	40.1% (1.7)	41.2% (1.4)	43.9% (1.2)	187.90b	.146	.99
	–	69.9% (5.4)^**^	76.6 % (10.4)^**^	62.4% (0.1)	58.4% (−3.2)	54.2% (−7.7)^**^	59.9% (−1.7)	58.8% (−1.4)	56.1% (−1.2)			
2. Suicidal ideation	+	1.6% (−5.0)^**^	2.1% (−4.3)^**^	5.1% (0.9)	4.0% (−1.3)	6.7% (4.3)^**^	6.8% (3.6)	6.5% (1.6)	2.4% (−1.0)	76.81b	.090	.99
	–	98.4% (5.0)^**^	97.9% (4.3)^**^	94.9% (−0.9)	96.0% (1.3)	93.3% (−4.3)^**^	93.2% (−3.6)	93.5% (−1.6)	97.6% (1.0)			
3. Anxiety	+	35.3% (−4.3)^**^	32.6% (−6.5)^**^	42.1% (0.4)	42.2% (0.5)	48.1% (6.1)^**^	45.0% (2.4)	41.2% (−0.2)	41.5% (0.0)	88.86b	.101	.99
	–	64.7% (4.3)^**^	67.4% (6.5)^**^	57.9% (−0.4)	57.8% (−0.5)	51.9% (−6.1)^**^	55.0% (−2.4)	58.5% (0.2)	58.5% (0.0)			
4. Irritability	+	23.6% (−7.6)^**^	18.2% (−12.0)^**^	31.1% (−3.3)^*^	36.4% (1.7)	43.4% (8.8)^**^	42.9% (6.4)^**^	47.4% (5.4)^**^	53.7% (3.7)^**^	327.99b	.196	.99
	–	76.4% (7.6)^**^	81.8% (12.0)^**^	68.9% (3.3)^*^	63.6% (−1.7)	56.6% (−8.8)^**^	57.1% (−6.4)^**^	52.6% (−5.4)^**^	46.3% (−3.7)^**^			
5. Aggression	+	9.1% (−7.3)^**^	7.6% (−9.1)^**^	13.5% (−4.9)^**^	17.8% (0.6)	23.4% (7.5)^**^	24.6% (6.9)^**^	31.8% (7.6)^**^	30.5% (3.2)^*^	288.19b	.183	.99
	–	90.9% (7.3)^**^	92.4% (9.1)^**^	86.5% (4.9)^**^	82.8% (−0.6)	76.6% (−7.5)^**^	75.4% (−6.9)^**^	68.2% (−7.6)^**^	69.5% (−3.2)^*^			
6. Apathy	+	8.6% (−17.1)^**^	9.3% (−17.4)^**^	19.8% (−13.0)^**^	29.7% (−2.3)	49.2% (16.5)^**^	60.3% (21.3)^**^	66.8% (14.5)^**^	77.4% (8.2)^**^	1542.24b	.424	.99
	–	91.4% (17.1)^**^	90.7% (17.4)^**^	80.2% (13.0)^**^	70.3% (2.3)	50.8% (−16.5)^**^	39.7% (−21.3)^**^	33.2% (−14.5)^**^	25.6% (−8.2)^**^			
7. Obsessive compulsive	+	7.3% (−13.4)^**^	6.5% (−14.7)^**^	12.8% (−13.1)^**^	20.8% (−3.4)	35.3% (11.6)^**^	50.2% (21.4)^**^	60.6% (16.6)^**^	54.9% (6.4)^**^	1304.79b	.390	.99
	–	92.7% (13.4)^**^	93.5% (14.7)^**^	87.2% (13.1)^**^	79.2% (3.4)	64.7% (−11.6)^**^	49.8% (−21.4)^**^	39.4% (−16.6)^**^	45.1% (−6.4)^**^			
8. Perseveration	+	3.4% (−10.2)^**^	6.8% (−7.1)^**^	10.1% (−5.2)^**^	13.8% (−0.1)	19.1% (7.0)^**^	22.3% (8.7)^**^	27.0% (7.5)^**^	36.6% (6.0)^**^	352.77b	.203	.99
	–	96.6% (10.2)^**^	93.2% (7.1)^**^	89.9% (5.2)^**^	86.2% (0.1)	80.9% (−7.0)^**^	77.7% (−8.7)^**^	73.0% (−7.5)^**^	63.4% (−6.0)^**^			
9. Paranoid thinking^a^	+	0.3% (−5.3)^**^	0.9% (−4.2)^**^	1.4% (−4.3)^**^	2.3% (−1.5)	4.6% (4.6)^**^	5.5% (5.5)^**^	8.4% (6.4)^**^	11.1% (4.4)^**^	152.87b	.136	.99
	–	99.7% (5.3)^**^	99.1% (4.2)^**^	98.6% (4.3)^**^	97.7% (1.5)	95.4% (−4.6)^**^	94.5% (−5.5)^**^	91.6% (−6.4)^**^	88.9% (−4.4)^**^			
10. Hallucinations^a^	+	0.1% (−3.0)^*^	0.6% (−1.2)	0.5% (−2.1)	0.1% (−3.7)^**^	1.0% (0.1)	1.8% (3.0)^*^	6.2% (10.5)^**^	4.9% (3.7)^**^	97.50b	.133	.99
	–	99.9% (3.0)^*^	99.4% (1.2)	99.5% (2.1)	99.9% (3.7)^**^	99.0% (−0.1)	98.2% (−3.0)^*^	93.8% (−10.5)^**^	95.1% (−3.7)^**^			
11. Disorientation^a^	+	0.4% (−11.5)^**^	0.9% (−11.4)^**^	2.1% (−13.9)^**^	4.0% (−9.1)^**^	13.9% (4.2)^**^	32.0% (23.8)^**^	53.6% (26.7)^**^	62.2% (14.8)^**^	1578.26b	.466	.99
	–	99.6% (11.5)^**^	99.1% (11.4)^**^	97.9% (13.9)^**^	96.0% (9.1)^**^	86.1% (−4.2)^**^	68.0% (−23.8)^**^	46.4% (−26.7)^**^	37.8% (−14.8)^**^			

*Note*: This type of analysis is not reported often. Consequently, to illustrate the analytic process, this note describes the apathy outcomes as an example. This table shows post hoc comparisons for apathy by subsample at administration time 1. The negatively adjusted residual z‐scores are seen to significantly differ from the null hypotheses for the family control, genotype‐negative, and premanifest subsamples, indicating significantly lower apathy from the null hypothesis. The positively adjusted residual z‐scores also significantly differ from the null hypotheses for the Stages 2–5 HD subsamples, indicating significantly higher apathy. Only the Stage 1 HD subsample did not significantly differ from the null hypothesis.

*w* ≥ .50 = large effect size, .3 ≤ *w* < .5 = moderate effect size, and .1 ≤ *w* < .3 = small effect size. + = symptom present; – = symptom not present.

^a^ Chi‐square and statistical significance computed from Fisher–Freeman–Halton exact test of independence (or Fishers exact test), due to small cell sizes less than 5. Fisher's exact test can be used when one of the cells in a table contains 0.

^b^ *p* < .01 (chi‐square analysis).

*p < .003125 (significant at the 95% confidence interval, Bonferroni correction adjusting for 16 analyses).

**p < .000625 (significant at the 99% confidence interval, Bonferroni correction adjusting for 16 analyses).

**TABLE 3:** Frequency (%), chi‐square, effect size, and post hoc comparison statistics for mental health symptom by subsample at Time 2 (*n* = 4644)

		Family controls	Genotype‐negative	Premanifest	Stage 1	Stage 2	Stage 3	Stage 4	Stage 5			
		*n* = 517	*n* = 546	*n* = 925	*n* = 659	*n* = 998	*n* = 706	*n* = 234	*n* = 59			
		Percentage of individuals showing presence of symptoms (adjusted residual *z*‐score)								*χ* ^2^	*w*	Power
1. Depression	+	28.6%(−3.6)	24.0%(−6.2)^**^	37.6%(1.2)	34.9%(−0.6)	41.7%(4.3)^**^	40.1%(2.5)	34.2%(−0.6)	50.8%(2.4)	72.93b	.128	.99
	–	71.4% (3.6)	76.0% (6.2)^**^	62.4% (−1.2)	65.1% (0.6)	58.3% (−4.3)^**^	59.9% (−2.5)	65.8% (0.6)	49.2% (−2.4)			
2. Suicidal ideation	+	1.7% (−3.2)^*^	1.3% (−3.8)^**^	5.5% (1.6)	4.1% (−0.5)	5.2% (1.3)	6.2% (2.5)	5.6% (0.8)	8.5% (1.5)	33.39b	.085	.99
	–	98.3% (3.2)^*^	98.7% (3.8)^**^	94.6% (−1.6)	95.9% (0.5)	94.8% (−1.3)	93.8% (−2.5)	94.4% (−0.8)	91.5% (−1.5)			
3. Anxiety	+	34.0% (−3.7)^**^	29.7% (−6.1)^**^	42.5% (0.6)	43.7% (1.1)	46.1% (3.2)^*^	47.0% (3.1)^*^	39.7% (−0.6)	54.2% (2.0)	66.64b	.120	.99
	–	66.0% (3.7)^**^	70.3% (6.1)^**^	57.5% (−0.6)	56.3% (−1.1)	53.9% (−3.2)^*^	53.0% (−3.1)^*^	60.3% (0.6)	45.8% (−2.0)			
4. Irritability	+	23.8% (−5.0)^**^	17.6% (−8.4)^**^	31.9% (−1.2)	35.4% (1.0)	38.9% (4.0)^**^	41.4% (4.8)^**^	44.4% (3.6)^**^	47.5% (2.3)	136.19b	.171	.99
	–	76.2% (5.0)^**^	82.4% (8.4)^**^	68.1% (1.2)	64.6% (−1.0)	61.1% (−4.0)^**^	58.6% (−4.8)^**^	55.6% (−3.6)^**^	52.5% (−2.3)			
5. Aggression	+	11.4% (−4.3)^**^	7.3% (−7.0)^**^	15.6% (−2.4)	18.8% (0.4)	21.1% (2.7)	26.5% (6.2)^**^	27.8% (3.9)^**^	28.8% (2.1)	120.89b	.161	.99
	–	88.6% (4.3)^**^	92.7% (7.0)^**^	84.4% (2.4)	81.2% (−0.4)	78.9% (−2.7)	73.5% (−6.2)^**^	72.2% (−3.9)^**^	71.2% (−2.1)			
6. Apathy	+	8.9% (−12.5)^**^	9.5% (−12.6)^**^	16.4% (−12.2)^**^	27.2% (−3.6)^**^	49.2% (12.0)^**^	61.3% (17.1)^**^	65.8% (10.8)^**^	71.2% (6.2)^**^	919.30b	.445	.99
	–	91.1% (12.5)^**^	90.5% (12.6)^**^	83.6% (12.2)^**^	72.8% (3.6)^**^	50.8% (−12.0)^**^	38.7% (−17.1)^**^	34.2% (−10.8)^**^	28.8% (−6.2)^**^			
7. Obsessive compulsive	+	4.4% (−12.0)^**^	6.6% (−11.1)^**^	11.8% (−11.2)^**^	21.9% (−2.8)	37.4% (9.0)^**^	51.7% (16.7)^**^	57.3% (11.1)^**^	59.3% (5.8)^**^	792.10b	.413	.99
	–	95.6% (12.0)^**^	93.4% (11.1)^**^	88.2% (11.2)^**^	78.1% (2.8)	62.6% (−9.0)^**^	48.3% (−16.7)^**^	42.7% (−11.1)^**^	40.7% (−5.8)^**^			
8. Perseveration	+	4.4% (−7.0)^**^	6.8% (−7.1)^**^	10.6% (−3.9)^**^	13.9% (−0.6)	19.3% (4.7)^**^	23.3% (7.1)^**^	24.2% (4.3)^**^	26.5% (2.8)	168.60b	.191	.99
	–	95.6% (7.0)^**^	93.2% (7.1)^**^	89.4% (3.9)^**^	86.1% (0.6)	80.7% (−4.7)^**^	76.7% (−7.1)^**^	75.8% (−4.3)^**^	73.5% (−2.8)			
9. Paranoid thinking^a^	+	0.0% (−4.0)^**^	0.9% (−2.7)	1.4% (−2.7)	3.0% (0.6)	3.1% (0.9)	5.5% (5.0)^**^	6.0% (3.2)^*^	5.1% (1.1)	66.20b	.114	.99
	–	100% (4.0)^**^	99.1% (2.7)	98.6% (2.7)	97.0% (−0.6)	96.9% (−0.9)	94.5% (−5.0)^**^	94.0% (−3.2)^*^	94.9% (−1.1)			
10. Hallucinations^a^	+	0.0% (−2.7)	0.4% (−1.9)	0.3% (−2.7)	0.3% (−2.3)	1.0% (−0.7)	3.0% (4.7)^**^	6.0% (6.9)^**^	6.8% (3.9)^*^	72.63b	.146	.99
	–	100.0% (2.7)	99.6% (1.9)	99.7% (2.7)	99.7% (2.3)	99.0% (0.7)	97.0% (−4.7)^**^	94.0% (−6.9)^**^	93.2% (−3.9)^*^			
11. Disorientation^a^	+	0.6% (−9.0)^**^	0.7% (−9.1)^**^	2.6% (−10.6)^**^	4.4% (−7.2)^**^	13.0% (−0.1)	35.1% (18.8)^**^	58.1% (20.9)^**^	59.3% (10.6)^**^	978.64b	.488	.99
	–	99.4% (9.0)^**^	99.3% (9.1)^**^	97.4% (10.6)^**^	95.6% (7.2)^**^	87.0% (0.1)	64.9% (−18.8)^**^	41.9% (−20.9)^**^	40.7% (−10.6)^**^			

*Note*: *w* ≥ .50 = large effect size, .3 ≤ *w* < .5 = moderate effect size, and .1 ≤ *w* < .3 = small effect size. + = symptom present; – = symptom not present.

^a^ Chi‐square and statistical significance computed from Fisher–Freeman–Halton exact test of independence (or Fishers exact test), due to small cell sizes less than 5. Fisher's exact test can be used when one of the cells in a table contains 0.

^b^ *p* < .01 (chi‐square analysis).

*p < .003125 (significant at the 95% confidence interval, Bonferroni correction adjusting for 16 analyses).

**p < .000625 (significant at the 99% confidence interval, Bonferroni correction adjusting for 16 analyses).

**TABLE 4:** Frequency (%), chi‐square, effect size, and post hoc comparison statistics for mental health symptom by subsample at Time 3 (*n* = 1845)

		Family controls	Genotype‐negative	Premanifest	Stage 1	Stage 2	Stage 3	Stage 4	Stage 5			
		*n* = 275	*n* = 245	*n* = 358	*n* = 227	*n* = 394	*n* = 244	*n* = 84	*n* = 18			
		Percentage of individuals showing presence of symptoms (adjusted residual *z*‐score)								*χ* ^2^	*w*	Power
1. Depression	+	25.5%(−2.3)^*^	20.8% (−3.8)^**^	32.4% (0.5)	34.8% (1.2)	36.8% (2.5)^*^	35.7% (1.6)	29.8% (−0.3)	27.8% (−0.3)	26.27b	.119	.98
	–	74.5% (2.3)^*^	79.2% (3.8)^**^	67.6% (−0.5)	65.2% (−1.2)	63.2% (−2.6)^*^	64.3% (−1.6)	70.2% (0.3)	72.2% (0.3)			
2. Suicidal ideation^a^	+	2.2% (−1.9)	0.8% (−2.9)	3.6% (−0.8)	2.6% (−1.4)	8.1% (4.1)^**^	6.1% (1.4)	7.1% (1.3)	5.6% (0.2)	29.86b	.126	.99
	–	97.8% (1.9)	99.2% (2.9)	96.4% (0.8)	97.4% (1.4)	91.9% (−4.1)^**^	93.9% (−1.4)	92.9% (−1.3)	94.4% (−0.2)			
3. Anxiety	+	32.0% (−2.2)	27.3% (−3.7)^**^	40.2% (1.0)	41.0% (1.0)	42.4% (2.0)	43.4% (1.9)	31.0% (−1.4)	55.6% (1.5)	28.02b	.123	.99
	–	68.0% (2.2)	72.7% (3.7)^**^	59.8% (−1.0)	59.0% (−1.0)	57.6% (−2.0)	56.6% (−1.9)	69.0% (1.4)	44.4% (−1.5)			
4. Irritability	+	21.5% (−3.3)^*^	13.1% (−6.2)^**^	28.5% (−0.6)	35.2% (1.9)	36.0% (3.0)^*^	36.6% (2.4)	44.0% (2.9)	55.6% (2.4)	71.78b	.197	.99
	–	78.5% (3.3)^*^	86.9% (6.2)^**^	71.5% (0.6)	64.8% (−1.9)	64.0% (−3.0)^*^	63.5% (−2.4)	56.0% (−2.9)	44.4% (−2.4)			
5. Aggression	+	10.5% (−2.5)	3.3% (−5.7)^**^	14.5% (−0.6)	18.1% (1.1)	20.3% (2.9)	20.1% (2.1)	26.2% (2.8)	33.3% (2.1)	56.93b	.176	.99
	–	89.5% (2.5)	96.7% (5.7)^**^	85.5% (0.6)	81.9% (−1.1)	79.7% (−2.9)	79.9% (−2.1)	73.8% (−2.8)	66.7% (−2.1)			
6. Apathy	+	8.0% (−8.7)^**^	7.3% (−8.4)^**^	21.5% (−4.0)^**^	31.3% (0.4)	44.4% (6.9)^**^	59.0% (10.5)^**^	50.0% (4.0)^*^	55.0% (1.8)	290.57b	.397	.99
	–	92.0% (8.7)^**^	92.7% (8.4)^**^	78.5% (4.0)^**^	68.7% (−0.4)	55.6% (−6.9)^**^	41.0% (−10.5)^**^	50.0% (−4.0)^*^	50.0% (−1.8)			
7. Obsessive compulsive	+	4% (−8.4)^**^	4.1% (−7.9)^**^	11.7% (−6.1)^**^	21.6% (−0.9)	38.8% (7.7)^**^	50.8% (10.5)^**^	56.0% (7.0)^**^	44.4% (2.0)	338.12b	.428	.99
	–	96.0% (8.4)^**^	95.9% (7.9)^**^	88.3% (6.1)^**^	78.4% (0.9)	61.2% (−7.7)^**^	49.2% (−10.5)^**^	44.0% (−7.0)^**^	55.6% (−2.0)			
8. Perseveration^a^	+	3.3% (−4.8)^**^	7.8% (−2.2)	8.1% (−2.5)	15.4% (1.7)	18.3% (4.3)^**^	17.2% (2.7)	16.7% (1.3)	11.1% (−0.1)	59.08b	.172	.99
	–	96.7% (4.8)^**^	92.2% (2.2)	91.9% (2.5)	84.6% (−1.7)	81.7% (−4.3)^**^	82.8% (−2.7)	83.3% (−1.3)	88.9% (0.1)			
9. Paranoid thinking^a^	+	1.1% (−0.6)	1.2% (−0.4)	1.4% (−0.2)	0.4% (−1.4)	1.3% (−0.5)	2.9% (1.9)	4.8% (2.5)	0.0% (−0.5)	9.461	.079	.69
	–	98.9% (0.6)	98.8% (0.4)	98.6% (0.2)	99.6% (1.4)	98.7% (0.5)	97.1% (−1.9)	95.2% (−2.5)	100.0% (0.5)			
10. Hallucinations^a^	+	0.0% (−1.4)	0.8% (0.6)	0.0% (−1.6)	0.0% (−1.2)	0.3% (−1.0)	2.9% (5.0)^**^	1.2% (0.7)	0% (0.3)	18.55b	.123	.99
	–	100.0% (1.4)	99.2% (−0.6)	100.0% (1.6)	100.0% (1.2)	99.7% (1.0)	97.1% (−5.0)^**^	98.8% (−0.7)	100% (−0.3)			
11. Disorientation	+	0.7% (−5.6)^**^	0.4% (−5.5)^**^	0.6% (−6.7)^**^	5.7% (−2.4)	12.4% (1.6)	29.1% (10.4)^**^	48.8% (11.9)^**^	55.6% (6.4)^**^	366.90b	.446	.99
	–	99.3% (5.6)^**^	99.6% (5.5)^**^	99.4% (6.7)^**^	94.3% (2.4)	87.6% (−1.6)	70.9% (−10.4)^**^	51.2% (−11.9)^**^	44.4% (−6.4)^**^			

*Note*: *w* ≥ .50 = large effect size, .3 ≤ *w* < .5 = moderate effect size, and .1 ≤ *w* < .3 = small effect size. + = symptom present; – = symptom not present.

^a^ Chi‐square and statistical significance computed from Fisher–Freeman–Halton exact test of independence (or Fishers exact test), due to small cell sizes less than 5 including 0. Fisher's exact test can be used when one of the cells in a table contains 0.

^b^ *p* < .01 (chi‐square analysis).

*p < .003125 (significant at the 95% confidence interval, Bonferroni correction adjusting for 16 analyses).

**p < .000625 (significant at the 99% confidence interval, Bonferroni correction adjusting for 16 analyses).

### 2.3 Data analysis

The analysis comprised two stages. First, we evaluate overall effects for the associations between the eight HD subsamples (variable 1; eight levels; categorical) and the presence or absence of each of the PBA‐s indices (variable 2; two levels; dichotomous/categorical). Second, we assess where the specific differences lie between the eight groups in terms of the presence or absence of each of the PBA‐s indices.

First, to assess overall effects, we report 33 chi‐square tests of association (Monte Carlo test based on 10,000 randomized samples with a 99% confidence interval), evaluating HD subgroup differences for 11 PBA‐s indices by three administration points, using Times 2 and 3 to assess the replicability of findings from Time 1. When the observed value for cells was less than 5, we report the chi‐square statistic and statistical significance using the Fisher–Freeman–Halton exact test (Freeman & Halton, 1951). As the accuracy of statistical significance level in chi‐square is vulnerable to sample size, we assessed the importance of the association using effect sizes for chi‐square (Kim, 2017). With smaller contingency tables, the tendency is to report the Cramer's *V* statistic as a measure of effect size. However, for larger contingency tables (e.g., ours is 8 [groups] × 2 [presence vs. absence of symptoms]) there is no standardized guidance on Cramer's *V*. Instead, Cohen's *w* can be calculated (*w* = Cramer's V × square root(*k* – 1), where *k* is the number of whichever is smaller of rows/columns) (Cohen, 1988; Sheskin, 2000). The magnitude of the effect size of *w* can be categorized with *w* ≥ .50 considered “large,” .3 ≤ *w* < .5 “moderate,” and .1 ≤ *w* < .3 “small” (Cohen, 1988; Sheskin, 2000). To ensure that overall associations were meaningful, we used the criterion of at least a moderate effect size (.3 ≤ *w* < .5). This is the minimum at which findings can be considered of practical significance (Cohen, 1988).

Second, to identify specific differences between the eight groups, we evaluated between‐group differences in PBA‐s symptom severity via post hoc comparisons between the eight HD subsamples for each chi‐square. We quantified between‐group differences based on variation from a null hypothesis of expected observations. This technique uses a multiple‐regression approach to analyze the differences between cells, using adjusted residual *z*‐scores for accounting for the overall sample size, and variation due to that sample size (Beasley & Schumacher, 1995; García‐Pérez & Núñez‐Antón, 2003). Typically, a *z*‐score of ±1.96 shows a statistically significant difference between cells at *p* < .05. Accordingly, we calculated a further chi‐square for each post hoc comparison by squaring the adjusted residual *z*‐score, and calculating the significance value using the SPSS 25 chi‐square function with 1 degree of freedom for each comparison of each group. As we conducted a large number of comparisons on data collected at the same timepoint (16 group comparisons across 11 PBA‐s indices, i.e., 176 comparisons at each timepoint), we computed a Bonferroni correction to assess significance for 176 analyses per timepoint and prevent type I error. The significance criteria were accordingly p < .00028409 for the 95% confidence interval (.05 divided by 16) and p < .00005682 for the 99% confidence interval (.01 divided by 16). Finally, we conducted a repeated measures analysis of variance as an additional marker of progress over time longitudinally. All analyses are reported in full for transparency, even if initial chi‐square statistics indicated a small effect size.

## 3 RESULTS

Tables 2, 3, 4 show the percentage of each mental health symptom present (+) and absent (–) by subsample, providing chi‐square, effect size, and post hoc comparison statistics (Table 2 shows Time 1; Table 3 shows Time 2; Table 4 shows Time 3). For apathy, obsessive–compulsive behaviors, and disorientation at each administration, a significant difference of medium effect was found (a minimum at which the findings are considered to be of practical significance).

The post hoc comparisons show a broad pattern of significantly higher and lower differences from the null hypothesis of expected observations. For both apathy and obsessive–compulsive symptoms, a significantly higher frequency from expected observations was seen across administrations for the Stages 2–5 HD groups. Furthermore, a significantly lower frequency of apathy and obsessive–compulsive symptoms was seen in the family controls, genotype‐negative, and premanifest groups across administrations (and at Stage 1 only for apathy). For disorientation, significantly higher frequency from expected observations was seen within the Stage 3–5 HD subsamples. Furthermore, broadly, where there was significantly lower frequency of disorientation from expected observations, these tended to be within family controls, genotype‐negative, premanifest, and Stages 1 and 2 of HD.

There are exceptions to these findings. Those with Stage 5 HD did not significantly differ from the expected observations for apathy and obsessive–compulsive symptoms at administration 3. Those with Stage 2 HD did not significantly differ in disorientation from expected observations at administration 1, and neither did those with Stage 1 or 2 HD at administration 3.

Finally, we considered changes in the presence of symptoms for the sample who provided responses across the three time periods using repeated measures analysis of variance. This method is an appropriate statistical technique for analyzing dichotomous outcome data where the degrees of freedom are greater than 40 (Lunney, 1970). Across the 11 PBA symptoms, there were no significant effects for 9 of the 11 symptom measures (*F* ≤ 1.42, df = 3328, *p* ≥ .207). However, there were significant main effects for obsessive–compulsive symptoms (*F* = 6.67, *p* < .001) and disorientation (*F* = 5.76, *p* = .003). In both cases, the differences were between Time 3 and Time 1 (both *p* < .002): obsessive–compulsiveness (Time 1, mean = .204, SD = .40; Time 3, mean = .244, SD = .43) and disorientation (Time 1, mean = .077, SD = .27; Time 3, *M* = .105, SD = .41).

## 4 DISCUSSION

In this study, we examined psychological symptoms via PBA‐s items between people at HD Stages 1–5, and HD family members who do not carry the gene expansion. This enabled evaluation of the frequency of symptoms throughout the disease course, and identification of symptoms that also occur in noncarriers (family controls and genotype‐negative) within HD families. Clarifying these differences has important implications for psychological interventions in pwHD, and for supporting other people within HD‐affected families.

Our findings reconfirm apathy as a common symptom in HD, which unlike other psychological symptoms (Paulsen et al., 2001; Van Duijn et al., 2013; Zappacosta et al., 1996) has been clearly linked to disease progression (Martinez‐Horta et al., 2016; van Duijn et al., 2014). In our study, people with later‐stage HD (Stages 2–5) typically showed higher levels of apathy, while the family control, genotype‐negative, and premanifest subsamples showed lower levels. Recent research has identified apathy to be associated with progressive atrophy of the thalamus among pwHD and loss of gray matter within the corticosubcortical network (Martínez‐Horta et al., 2018), indicating a neural basis for the higher prevalence of apathy in later‐stage HD.

People with later‐stage HD (2–5) also had higher obsessive–compulsive symptoms than the premanifest and two noncarrier groups (family controls and genotype‐negative). This suggests that obsessive–compulsive behaviors are also associated with HD progression, potentially related to increasing orbitofrontal and striatal dysfunction over time (Anderson et al., 2011; Nakao et al., 2014; Oosterloo et al., 2019). Consideration of the potential role of medication and comorbidities may be important when judging the relevance of obsessive–compulsive behaviors in HD, however, as such symptoms may result from these factors rather than from progression. It is also clinically important to consider the difference between obsessive–compulsive behaviors and perseverative behaviors stemming from executive function changes (Oosterloo et al., 2019). While these may appear externally similar (repetitive behaviors), clearly potential treatment routes would differ depending on cause.

Finally, disorientation was also elevated for the later stages of the manifest subsample (predominantly stages 3–5), highlighting that this symptom may emerge later in the disease course than obsessive–compulsive behaviors and apathy, as cognition deteriorates significantly. Disorientation emerging as a key symptom in HD progression is concordant with prior findings of apathy, disorientation, and perseveration together forming an apathy subscale for the PBA‐s (Callaghan et al., 2015). The neurological basis of disorientation generally appears less well‐studied than the basis of apathy and obsessive–compulsive symptoms. However, one recent study (Lemoine et al., 2021) linked temporal disorientation with cell atrophy in the striatum (caudate and putamen), suggesting differential progression of HD‐related disorientation with greater temporal than spatial difficulties emerging over time. This provides one potential neurobiological mechanism for the differentiation between the manifest and three remaining subsamples.

Importantly, significant differences in symptom frequency between pwHD (manifest and premanifest) and noncarriers (family controls and genotype‐negative) were only found for three indices of the PBA‐s, and not for the remaining eight (depression, suicidal ideation, anxiety, irritability, aggression, perseveration, paranoia, and hallucinations). This supports previous findings that a degree of psychological distress in various forms is shared between carriers and noncarriers across HD‐affected families (Achenbach & Saft, 2021; Maltby et al., 2021). Descriptions of psychosocial contributions to HD‐related distress may support this interpretation, as both carriers and noncarriers may experience intense anxiety and distress relating to changed familial narratives, disrupted interpersonal dynamics, grief, and lost plans for the future (Berrios et al., 2002; Brouwer‐DudokdeWit et al., 2002; Tibben et al., 1997; Yu et al., 2019). Our findings demonstrate that some key clinical psychological symptoms of HD are shared to a similar degree by nonmanifest groups, concordant with these previous findings.

### 4.1 Clinical implications and future research

These findings highlight key psychological changes as HD progresses, and demonstrate the importance of considering apathy, disorientation, and obsessive–compulsive symptoms in the clinical management of pwHD. There is a clear need for strategies to mitigate against the potential negative physical health and psychological effects of these difficulties. Further research into the neurological basis of disorientation and obsessive–compulsive behaviors in HD also appears critical.

The identification of similarities as well as differences between people with manifest HD and the premanifest, family control, and genotype‐negative groups carries important clinical implications for supporting families affected by HD. There appears to be a shared level of distress across eight PBA‐s domains, which promotes the importance of considering systemic well‐being in the treatment of HD, and potentially considering family‐level interventions or some difficulties. Maintenance of carer well‐being is also crucial due to the key role they play in supporting pwHD. Future research might explore potential systemic contributions to HD‐related distress, alongside the more familiar impacts of physical and cognitive changes.

### 4.2 Strengths and limitations

We used a large international data set and established measures with strong psychometric properties to evaluate specific clinical symptoms relating to psychological well‐being in pwHD across the disease course, and in comparison to additional HD‐related populations. This enabled us to both understand HD symptoms as the condition progresses and evaluate psychological difficulties found across HD families. We also examined these data across three timepoints and the findings evidenced high stability in our large sample, indicating robust results.

In terms of limitations, we did not examine data regarding medication use among participants—as noted, this may be of particular relevance regarding obsessive–compulsive symptoms. However, our findings concur with a robust literature supporting apathy, obsessive–compulsiveness, and disorientation as key psychological symptomatology in HD, including proposed neurobiological mechanisms. It is also challenging to measure psychological symptoms in HD; worsening anosognosia over time can impair validity of self‐report (Gunn et al., 2020), and a lack of professional consensus around some mental health constructs, such as irritability, may reduce interrater reliability (Simpson et al., 2019). However, the consistency of our findings across timepoints and the consistency with other psychometric and neurobiological findings demonstrate an empirical consensus.

Importantly, those with the most advanced HD and poorest psychological well‐being are unlikely to be well‐represented in the Enroll‐HD data set, due to access issues, deterioration in motivation, and escalating cognitive and behavioral difficulties associated with the later stages that complicate or prevent research participation. Additionally, those attending clinics regularly for monitoring are likely to receive earlier and more consistent support. Consequently, those with better psychological well‐being and more support may be better represented. However, this would likely reduce rather than enhance differences between the manifest group and the other subsamples, so this does not detract from the reported findings but raises the possibility that for some, the identified difficulties may arise earlier in the disease course than indicated by our results.

Finally, it was not possible to meaningfully compare differences between mental health symptoms among people in HD‐affected families and controls from non‐HD families, because the latter are not available in the data set (and generic prevalence data may not adequately take into account key demographic variables known to contribute to well‐being). However, these comparisons will be crucial to demonstrate specific impacts of living in HD‐affected families on people who do not carry the gene, compared to equivalent groups not affected by HD. This is an important subject for future study, although beyond the scope of the current analysis.

## 5 CONCLUSION

In conclusion, this study identifies distinctive mental health symptoms among people with later‐stage manifest HD, with apathy and obsessive–compulsive behaviors emerging around stage 2 and disorientation around stage 3. This highlights important targets for clinical monitoring and intervention as HD progresses, as well as identifying potential psychological symptoms experienced in other members of HD families.

## AUTHOR CONTRIBUTIONS

**Sarah Gunn**: Writing—original draft (lead); writing—review and editing (lead); data interpretation and conceptualization (support). **Maria Dale**: Conceptualization; funding acquisition; writing—review and editing (support). **Noora Ovaska‐Stafford**: Data curation. **John Maltby**: Conceptualization; formal analysis and interpretation (lead); funding acquisition; writing—original draft; writing—review and editing.

## CONFLICT OF INTEREST STATEMENT

The authors declare no conflicts of interest.

### PEER REVIEW

The peer review history for this article is available at https://publons.com/publon/10.1002/brb3.2954.

## Data Availability

The data that support the findings of this study are available from Enroll‐HD (Enroll‐HD, RRID:SCR_023300). Restrictions apply to the availability of these data, which were used under license for this study.
